# Protective Effects of L-2-Oxothiazolidine-4-Carboxylate during Isoproterenol-Induced Myocardial Infarction in Rats: In Vivo Study

**DOI:** 10.3390/life12101466

**Published:** 2022-09-21

**Authors:** Marija Angelovski, Nikola Hadzi-Petrushev, Dino Atanasov, Aleksandar Nikodinovski, Vadim Mitrokhin, Dimiter B. Avtanski, Mitko Mladenov

**Affiliations:** 1 Institute of Biology, Faculty of Natural Sciences and Mathematics, Ss. Cyril and Methodius University in Skopje, 1000 Skopje, North Macedonia; 2 Institute for Preclinical and Clinical Pharmacology and Toxicology, Medical Faculty, Ss. Cyril and Methodius University in Skopje, 1000 Skopje, North Macedonia; 3 Department of Fundamental and Applied Physiology, Russian National Research Medical University, 117997 Moscow, Russia; 4 Friedman Diabetes Institute, Lenox Hill Hospital, Northwell Health, 110 E 59th Street, New York, NY 10022, USA

**Keywords:** 2-oxothiazolidine-4-carboxylic acid, isoproterenol, myocardial infarction, oxidative stress, inflammation

## Abstract

This study aimed to evaluate the cardioprotective effects of L-2-oxothiazolidine-4-carboxylate (OTC) against isoproterenol (ISO)-induced acute myocardial infarction (MI) in rats. Results demonstrated that OTC treatments inhibited ISO-induced oxidative damage, suppressed lipid peroxidation, and increased superoxide dismutase and catalase activity in the hearts of the treated rats compared to those of the untreated controls. The ISO-related NF-κB activation was reduced due to the OTC treatment, and lower degrees of inflammatory cell infiltration and necrosis in the hearts were observed. In summary, OTC treatments exerted cardioprotective effects against MI in vivo, mainly due to enhancing cardiac antioxidant activity.

## 1. Introduction

The critical misbalance between oxygen demand and supply in the myocardial tissue causes myocardial ischemia and, subsequently, myocardial infarction (MI). MI remains the leading cause of global mortality despite the rapid progression in strategies for treating myocardial ischemia [1].

At low concentrations, catecholamines are important regulators of myocardial contractility and metabolism. Still, excessive endogenous release or exogenous administration causes severe stress within the cardiac muscle, impaired energy metabolism, and biochemical and structural changes resulting in infarct-like cardiac tissue necrosis. Hence, the administration of the synthetic β-adrenoceptor agonist isoproterenol (ISO) in animals provides a rapid and non-invasive method that mimics the clinical conditions of MI in humans [2,3]. The underlying mechanisms of ISO-induced MI are complex and multifactorial but generally associated with generating cytotoxic reactive oxygen species (ROS) in cardiomyocytes, followed by oxidative stress [2,4,5]. This leads to progressive damage relative to the mitochondria and the intracellular accumulation of Ca^2+^, while oxidative stress also contributes to developing cardiac remodeling and fibrosis [6]. Additionally, MI triggers an inflammatory response that potentiates myocardial injuries in the early phase and is characterized by the excessive production of inflammatory cytokines and the recruitment of leukocytes into the ischemic area [7]. These processes are activated by the presence of ROS, which stimulates signal transduction and modulates the activity of specific transcription factors, including the NF-κB [7,8].

Previous studies have shown that during the development of MI, the heart has a limited antioxidant capacity, making it particularly vulnerable to ROS [9]. The cardiac glutathione (GSH)/glutathione peroxidase (GPx) system has a central role in ROS neutralization [10]. Therefore, maintaining adequate levels of GSH and improving the endogenous antioxidant capacity could be beneficial during the development of MI [11]. Both in vitro and in vivo studies demonstrated that increasing the endogenous GSH via *N*-acetylcysteine treatment abates oxidative stress and improves the outcome of MI [12,13,14,15]. *L*-2-oxothiazolidine-4-carboxylic acid (OTC), also known as procysteine, is an effective precursor of cysteine, the rate-limiting amino acid in GSH synthesis. OTC is a 5-oxo-*L*-prolinase substrate characterized by high membrane permeability and good systemic bioavailability that possess oxidative stress-protective [16,17,18,19] and anti-inflammatory activities [20,21]. In previous studies, OTC treatments demonstrated a lack of adverse effects and protective actions on the heart following injury [16,17]. To the best of our knowledge, this is the first in vivo study that evaluates the cardioprotective effects of OTC in the setting of MI in rats. The effects of OTC were evaluated by assessing the levels of endogenous antioxidants, lipid peroxidation and protein oxidation markers, the NF-κB transcriptional activity, and the histopathological alterations in the heart tissue.

## 2. Materials and Methods

### 2.1. Animals and Experimental Design

This study was approved by the Animal Ethics Committee of the Ss. Cyril and Methodius University in Skopje, Republic of Macedonia, following the International Guiding Principles for Biomedical Research Involving Animals. All experimental procedures were conducted according to the ethical guidelines approved by the Macedonian Center for Bioethics. The study was carried out utilizing 32 male Wistar rats (200–250 g b.w.). The animals were maintained under standard conditions of ventilation, temperature (22 ± 2 °C), relative humidity (45 ± 15%), light:dark cycle (12:12 h), and fed with standard rat chow and water ad libitum. Anesthetics were applied according to EC Directive 86/609/EEC guidelines.

All animals were randomly divided into four groups of 8 rats, depending on the application of ISO and/or OTC: control group (C), ISO-injected rats (ISO), OTC-treated rats (OTC), and ISO-injected and OTC-treated rats (ISO+OTC). ISO (100 mg/kg b.w.) was dissolved in saline and subcutaneously injected twice at an interval of 24 h to induce MI (13). Two hours after the second ISO injection, peripheral blood was collected to determine the cTn-I blood plasma level. OTC (Sigma, St. Louis, MO, USA) was dissolved in physiological saline, and the pH of the solution was adjusted to 7.0 with NaOH.

The rats in the OTC and ISO+OTC groups were treated with a total of 5.5 mmol/kg b.w. OTC divided into equal doses twice a day for two consecutive days, two hours before and two hours after the ISO injection. The rats in the control group (C) received vehicles only. Twenty-four hours after the last ISO injection, the rats from all experimental groups were anesthetized with thiopental sodium, 50 mg/kg b.w., i.p. Immediately after the sacrifice, the heart tissue was excised and washed in ice-cold saline. Part of the heart tissue, which included the base of the left ventricle, was fixed in 10% buffered formalin for histopathological analyses, while the other tissue pieces were snap-frozen in liquid nitrogen and stored at −80 °C until further analyzed.

### 2.2. Biochemical Estimations

The blood plasma level of the diagnostic marker cardiac troponin I (cTn I) was determined using a commercial ELISA kit (Rat TNNI3/cTn-I (Troponin I Type 3, Cardiac), Elabscience, Houston, TX, USA). The assays for the determination of tissue malondialdehyde (MDA) and advanced oxidation products of protein (AOPP) levels were performed according to the methods described by Ohkawa et al. [22] and Taylor et al. [23], respectively. The supernatants from the tissue homogenates were used for the estimation of enzymatic activities of superoxide dismutase (SOD) [24], catalase (CAT) [25], glutathione peroxidase (GPx) [26], and glutathione reductase (GR) [27]. Total reduced glutathione (GSH) levels in the heart tissue were estimated by the enzymatic recycling method described by Rahman et al. [28].

### 2.3. Histopathological Analysis

The fixed heart tissues were embedded in paraffin, and 5 μm-thick tissue sections from the base of the left ventricle were stained with hematoxylin and eosin (H&E) for examining histoarchitectural changes. Masson’s trichrome staining was used to differentiate necrotic from viable myocardium. The stained sections were analyzed using a light microscope.

### 2.4. RT-qPCR

The transcriptional activity of NF-κB was quantified according to the method of Bottero et al. [29]. Total RNA from frozen heart tissues was isolated using a spin column kit (GeneJET RNA Purification Kit, Thermo Fisher Scientific, Waltham, MA, USA). cDNA was generated using a TaqMan Reverse Transcription Reagents Kit (Applied Biosystems, Waltham, MA, USA). qPCR amplification was performed utilizing SYBR Green PCR Master Mix (Thermo Fisher Scientific) on the StepOne RT-PCR system (Applied Biosystems). The mRNA expression levels of IκB-α were assessed in relation to the expression levels of the housekeeping gene glyceraldehyde-3-phosphate dehydrogenase (GAPDH) using ΔΔCt analysis. PCR amplification was performed using the following primers: IκB-α, F: 5′-GGAAGATGAGTTGCCCTACGA-3′, R: 5′-CTGTGTGCTGTGGTGCTAAG-3′; GAPDH, F: 5′-TGAACGGGAAGCTCACTGG-3′, R: 5′-GCTTCACCACCTTCTTGATGTC-3′.

### 2.5. Statistical Analysis

Data on the graphs are expressed as mean ± standard error of the mean (SEM). Statistical analyses were performed by one-way analysis of variance using GraphPad Prism 4.0 (Prism GraphPad Software, San Diego, CA, USA). Tukey’s post hoc test was applied in selected instances to evaluate additional differences between group pairs. A value of *p* < 0.05 was considered statistically significant.

## 3. Results

### 3.1. Biomarker for Myocardial Infarction

**Figure 1:**
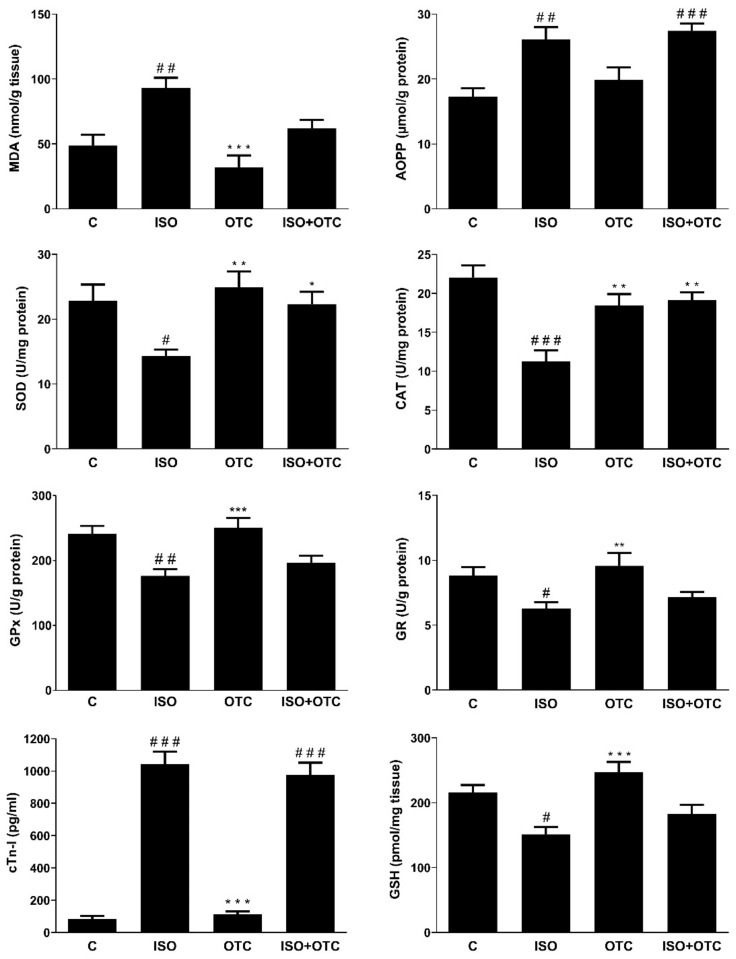
Effects of OTC treatment on the cTnI blood plasma levels, antioxidant enzyme activity, and levels of oxidative stress markers in the cardiac tissue. C—Control group; ISO—ISO-injected rats; OTC—OTC-treated rats; ISO+OTC—ISO and OTC-treated rats. ^#^ *p* < 0.05, ^##^ *p* <0.01, ^###^ *p* < 0.001—compared to C; * *p* < 0.05, ** *p* < 0.01, *** *p* < 0.001—compared to ISO.

The acute administration of ISO led to a significant increase in the cardiac injury biomarker, cTnI (Figure 1, C vs. ISO, *p* < 0.001). The OTC treatment did not affect the ISO-induced increase in cTnI levels (Figure 1, C vs. ISO+OTC, *p* < 0.001), and there was no significant difference in cTnI blood plasma levels between the ISO and the ISO+OTC groups (Figure 1). The OTC control group had normal levels of cTnI.

### 3.2. Antioxidant and Oxidative Stress Markers and NF-κB Transcriptional Activity

The induction of MI was associated with a decrease in the antioxidant capacity, leading to an elevation in the levels of oxidative stress markers and the transcriptional activity of the pro-inflammatory NF-κB (Figure 1 and Table 1). Compared to the controls, the rats in the ISO group had lower SOD (*p* < 0.05), CAT (*p* < 0.001), GPx (*p* < 0.01), and GR (*p* < 0.05) activities in the heart, as well as decreased GSH levels (*p* < 0.05). The decreased antioxidant capacity contributed to the development of oxidative stress in the cardiac tissue, as indicated by the significantly increased MDA and AOPP levels in the ISO group of rats compared to the controls (*p* < 0.01). In addition, as shown in Table 1, significantly higher NF-κB transcriptional activity, based on the expression of IκB-α, was detected in the cardiac tissue of the ISO group of rats compared to controls (*p* < 0.05).

Relative to the control, the OTC treatment had no effects on the analyzed markers in the rats without MI (Figure 1 and Table 1, OTC group). Conversely, OTC treatments successfully ameliorated some of the ISO-induced deleterious changes in the cardiac tissue of the rats with MI. GPx and GR activity, levels of GSH, and the transcriptional activity of NF-κB in the heart of rats were not significantly different between the ISO+OTC and the control group. Additionally, SOD (*p* < 0.05) and CAT (*p* < 0.01) activities in the hearts were significantly higher in the ISO+OTC compared to the ISO group. The improved antioxidant status was associated with a reduced level of lipid peroxidation, as indicated by the lack of significant difference in the cardiac MDA concentration between the ISO+OTC and the control rats. However, the OTC treatment in the rats with MI did not inhibit the ISO-induced increase in AOPP—a marker of protein oxidation (ISO+OTC vs. C, *p* < 0.001).

### 3.3. Histopathological Changes

**Figure 2:**
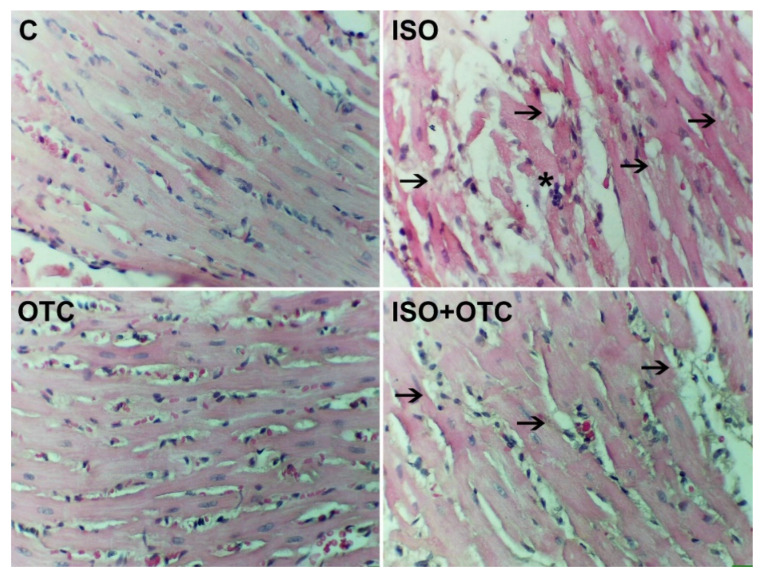
Representative micrographs of H&E-stained fields (40× magnification) of the left ventricles. C—Control group; ISO—ISO-injected rats; OTC—OTC-treated rats; ISO+OTC—ISO and OTC-treated rats. The asterisk indicates inflammatory cell infiltration, and the arrows point to examples of areas with muscle fiber rupture and the loss of myofibrils. The ISO group showed the most severe damage characterized by a disarrangement of fibers, fiber rupture, and necrosis.

The H&E-stained sections of cardiac tissue from control rats showed normal architecture without any sign of damage, i.e., normal nearby myofibrils with characteristic striations and continuity (Figure 2). The heart tissue of the rats from the ISO group showed significant inflammatory cell infiltration and a massive loss of myofibrils with marked necrosis. As shown on the representative micrograph of a heart slice from the rats in the ISO+OTC group, ISO-related histological alterations were moderately diminished under the influence of OTC treatments. This was evidenced by the reduced leukocyte infiltration and the reduced occurrence of cardiac muscle fibers rupture. The histomorphology of the heart tissue in the OTC group was comparable to the control group.

**Figure 3:**
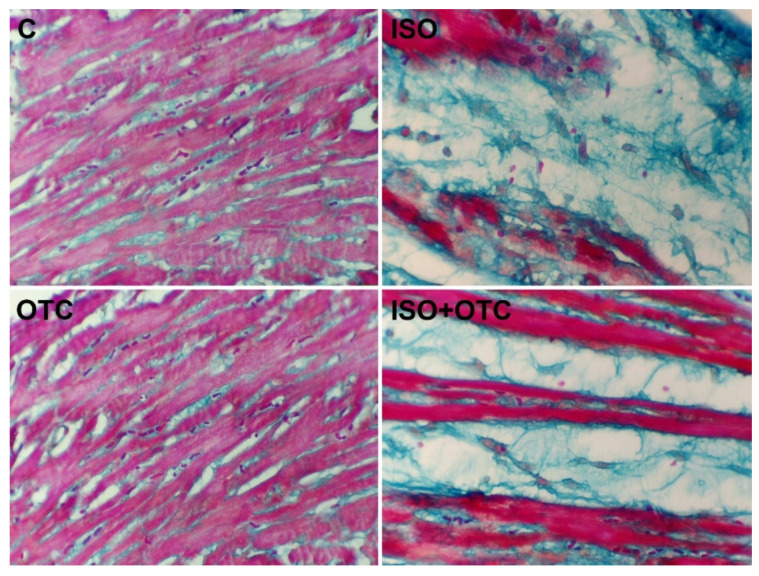
Representative micrographs of Masson’s trichrome-stained fields (40× magnification) of the left ventricles. C—Control group; ISO—ISO-injected rats; OTC—OTC-treated rats; ISO+OTC—ISO and OTC-treated rats. Decreased areas of viable myocardium (red) was associated with the application of ISO. The blue color (including large unstained areas) indicates necrotic myocardium.

The microscopic analysis of Masson’s trichrome-stained myocardial tissue sections showed the presence of healthy, red-colored tissue in the samples from the control and OTC groups (Figure 3). ISO-induced necrotic, blue-colored areas and significantly reduced areas of viable tissue were clearly evident in the samples from ISO-administered rats, indicating acute MI. When visually comparing the slides from the ISO and the ISO+OTC groups, it was apparent that OTC treatments partially diminished the size of blue-colored necrotic myocardium but did not prevent necrosis of the left ventricular tissue.

## 4. Discussion

Ischemic heart disease, ultimately progressing to MI, is one of the leading causes of death; therefore, there is a constant demand to explore novel therapeutic approaches. This study examined the effects of OTC treatments in the settings of the MI animal model.

The observed significant elevation in the blood plasma levels of the diagnostic marker for myocardial cell necrosis (cTnI) confirmed the initiation of MI in the rats. These findings were consistent with those obtained in other studies [3,4]. The rise in cTnI levels could be attributed to ISO-induced myocardial damage. The auto-oxidation of ISO, resulting in the generation of highly cytotoxic free radicals and subsequent oxidative stress, is the primary mechanism that leads to functional and structural cardiac muscle injury [4,30]. The animals treated with OTC did not show a significant reduction in the ISO-induced increase in cTnI levels, indicating that the occurrence of MI cannot be prevented by OTC, at least in the manner and dosage applied in this study. 

In the experimental model of MI, peroxidative damage is mainly responsible for the myocardial necrosis observed in ISO-injected rats [30]. This situation is precipitated by simultaneously reduced antioxidant defenses and increased ROS production, leading to a state of oxidative stress and inflammation. It is known that increased ROS production during heart failure is accompanied by reduced antioxidant mechanisms, i.e., decreased activities of SOD, CAT, and GPx, as well as the activation of the pro-inflammatory NF-κB [31,32]. These findings were corroborated by our results. Significantly decreased activities of SOD and CAT following ISO administration could be related to their utilization for ROS scavenging. Studies have shown that superoxide radicals modulate the activity of CAT and SOD, resulting in the loss of activity and accumulation of the superoxide radicals, which damages the myocardium [33]. Furthermore, the myocardial GSH levels were significantly reduced in ISO-injected rats, which concord with previously reported data [33,34] and suggests the increased utilization of the protective thiol-containing proteins by the lipid peroxides and the ROS. The decreased availability of reduced glutathione might also be a cause for the decreased activity of both GPx and GR in the myocardium of the ISO-injected rats [35,36]. Therefore, the observed significant increase in MDA and AOPP levels in the heart tissue of ISO-injected rats and the histopathological findings confirmed the presence of oxidative stress, which is characteristic of MI.

The OTC treatment led to a reduction in the ISO-induced lipid peroxidation in the heart tissue, possibly by decreasing oxidative stress. However, the AOPP levels in the heart tissue were not reduced in the function of OTC treatments, and the level of myocardial GSH was not significantly different between treated and untreated rats with MI. During heart failure, 5-oxoprolinase expression is reduced [37], which may lead to decreased conversion of OTC to cysteine. This might be one reason for the lack of a significant GSH increase in the myocardial tissue from the OTC-treated rats observed in our experiment. However, the OTC treatment did alleviate the ISO-induced changes in the cardiac GSH levels and in the activity of antioxidant enzymes, possibly by the extensive utilization of de novo-synthesized GSH and the neutralization of ISO-induced lipid peroxides and superoxide anions. Thus, the increased utilization of GSH could be related to GSH taking over the role of SOD and CAT in the early stages of MI, leading to decreased infarct size and improved cardiac tissue survival and function. In this context, Priscila and Prince [38] demonstrated that depressed GSH levels may be associated with an enhanced protective mechanism against oxidative stress in MI and improved recovery after a short period of ischemia.

Considering the histopathological findings in the heart tissue of treated rats, the results from our study suggested that OTC supplementation is sufficient for ameliorating some of the effects of the infarct injury in the model of ISO-induced MI. This was due to maintaining the level of GSH and the activity of the antioxidant enzymes in the heart, and possibly other properties of OTC, such as its anti-inflammatory activities [20,21]. In our experiment, ISO-mediated oxidative stress led to myonecrosis, a separation of cardiac muscle fibers, and characteristic leukocyte infiltration. This was in accordance with previous findings where activated NF-κB triggered the gene expression of interstitial and vascular adhesion molecules and monocyte chemoattractant protein-1 (MCP-1), leading to leukocyte infiltration into the infarcted myocardium [39]. We also found that OTC treatment suppressed NF-κB activity, which was beneficial, as evident from the markedly suppressed leukocyte infiltration and predominantly preserved integrity of the myofibrils in the tissue sections from the hearts of treated rats. In acute MI, the increasingly generated ROS stimulate signal transduction, leading to the production of inflammatory cytokines. In turn, the inflammatory cytokines regulate cell survival and cell death and also stimulate ROS formation [40]. For example, this can be achieved through stress-/ROS-sensitive mitogen-activated protein kinases that activate NF-κB. While this inflammatory reaction in the post-MI chronic stage contributes to the tissue repair of the injured myocardium, in acute MI, the effects of NF-κB-induced inflammatory cytokines are detrimental [7]. It has been previously shown that the reduced NF-κB activity is related to decreased oxidative stress in the myocardium [36,41]. Our findings were also consistent with the results of Lee et al. [42], which demonstrated that OTC inhibits the NF-κB signal transduction pathway by decreasing NF-κB binding activity relative to the promoter regions of genes that are involved in inflammation. Hence, we believe that the moderate inhibitory effect on the infarct injury due to the OTC treatment in our experiment was related to the decreased oxidative stress and the lowered NF-κB binding activity in the heart tissue.

Despite considerable advances in interventions and treatment, there is a high frequency of mortality in patients with MI. In the clinical setting, myocardial infarction is often complicated by cardiogenic shock and accompanied by acute kidney injury, which significantly contributes to mortality [43,44]. In these situations, inflammatory and redox status parameters are increasingly being taken into consideration because of their predictive value for the outcome [44]. Thus, potential therapeutic agents such as OTC, with the capacity to influence these parameters in the heart, as shown in this study, but also in other organs (e.g., the kidneys) [41], may prospectively lead to new therapeutic strategies.

## 5. Conclusions

In conclusion, the presented biochemical and histopathological findings confirmed the important role of oxidative stress and inflammation in the pathogenesis of MI. Our results show that OTC treatment has beneficial effects on MI by enhancing cardiac antioxidant mechanisms and reducing the activity of the pro-inflammatory NF-κB. This leads to a reduction in lipid peroxidation, thus preserving the integrity of heart tissue. The results from this study demonstrated the cardio-protective effects of OTC in the setting of MI in rats in vivo, prompting further research to investigate the molecular mechanisms of OTC’s action and the plausibility of its use in cases of ischemic heart disease.

## Figures and Tables

**Table 1:** Changes in the expression of IκB-α as an indicator of NF-κB’s transcriptional activity. Data is based on five biological replicates per group: C, control group; ISO, ISO-injected rats; OTC, OTC-treated rats; ISO+OTC, ISO, and OTC-treated rats.

Group	ΔCt (Mean ± SEM)	Fold Change (Normalized Relative to C)
C	9.071 ± 0.413	1 (0.751–1.332)
ISO	6.516 ± 0.710 ^1^	5.877 (3.592–9.617)
OTC	8.783 ± 0.908	1.221 (0.650–2.291)
ISO+OTC	7.972 ± 0.575	2.141 (1.438–3.189)

^1^ *p* < 0.05—compared to C.

## Data Availability

Data available on request to the corresponding author.
